# SPEECHLESS Speaks Loudly in Stomatal Development

**DOI:** 10.3389/fpls.2020.00114

**Published:** 2020-02-21

**Authors:** Liang Chen, Zhongliang Wu, Suiwen Hou

**Affiliations:** Key Laboratory of Cell Activities and Stress Adaptations, Ministry of Education, School of Life Sciences, Lanzhou University, Lanzhou, China

**Keywords:** SPCH, stomatal development, stomatal lineage, stomatal patterning, stomatal differentiatation

## Abstract

Stomata, the small pores on the epidermis of plant shoot, control gas exchange between the plant and environment and play key roles in plant physiology, evolution, and global ecology. Stomatal development is initiated by the basic helix-loop-helix (bHLH) transcription factor SPEECHLESS (SPCH), whose central importance in stomatal development has recently come to light. SPCH integrates intralineage signals and serves as an acceptor of hormonal and environmental signals to regulate stomatal density and patterning during the development. SPCH also plays a direct role in regulating asymmetric cell division in the stomatal lineage. Owing to its importance in stomatal development, *SPCH* expression is tightly and spatiotemporally regulated. The purpose of this review is to provide an overview of the SPCH-mediated regulation of stomatal development, reinforcing the idea that SPCH is the central molecular hub for stomatal development.

## Introduction

**Figure 1:**
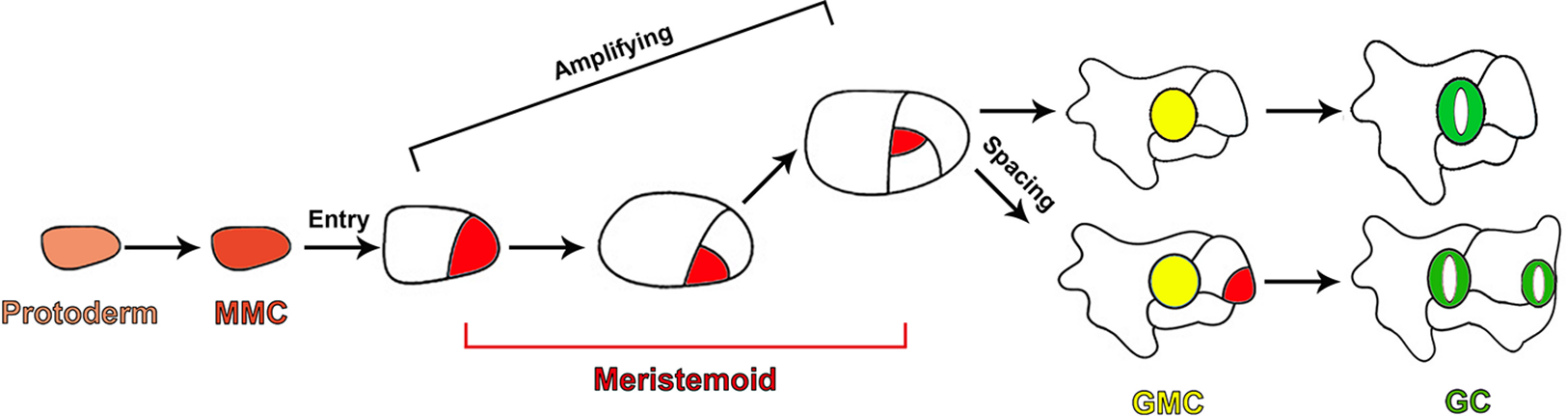
Diagram depicting cell fate transitions during the stomatal development in *Arabidopsis*. A subset of protodermal cells (faint red) acquire the fate of an MMC (brick red) and undergo asymmetric entry division, producing a meristemoid (red) and SLGC (white). Meristemoids undergo asymmetric amplifying divisions to increase the number of SLGCs while also self-renewing. Eventually, meristemoids differentiate into GMCs (yellow). Each GMC symmetrically divides to yield a pair of highly specialized GCs (green). SLGCs can also initiate stomatal development through oriented asymmetric spacing divisions.

In *Arabidopsis*, stomata formation depends on a series of cell divisions and consecutive cell fate transitions, producing five major cell types of the stomatal lineage, including meristemoid mother cells (MMCs), meristemoids, stomatal lineage ground cells (SLGCs), guard mother cells (GMCs), and guard cells (GCs) (Nadeau and Sack, 2002b; Bergmann and Sack, 2007; Lau and Bergmann, 2012; Pillitteri and Torii, 2012; Pillitteri and Dong, 2013). A subset of protodermal cells in the epidermis acquire the fate of MMCs and initiate the stomatal lineage by undergoing asymmetric entry divisions to produce the small triangular meristemoids and larger sister cells called SLGCs (**Figure 1**). Meristemoids carry out a limited number of asymmetric amplifying divisions to increase the number of SLGCs, while also performing the process of self-renewal (**Figure 1**). Finally, meristemoids lose their ability of reiterative asymmetric division and differentiate into GMCs. Each GMC symmetrically divides to yield a pair of highly specialized GCs (**Figure 1**) (Nadeau and Sack, 2002b; Bergmann and Sack, 2007; Lau and Bergmann, 2012; Pillitteri and Torii, 2012; Pillitteri and Dong, 2013). SLGCs can also acquire the MMC fate and undergo asymmetric division to produce satellite meristemoids that are oriented away from preexisting stomata or precursors. This asymmetric division, which prevents the direct contact between two stomata, is termed “oriented asymmetric spacing divisions”. Alternatively, SLGCs can terminally differentiate into pavement cells (**Figure 1**) (Geisler et al., 2000; Bergmann and Sack, 2007).

## Speechless (SPCH) Initiates the Stomatal Lineage

**Figure 2:**
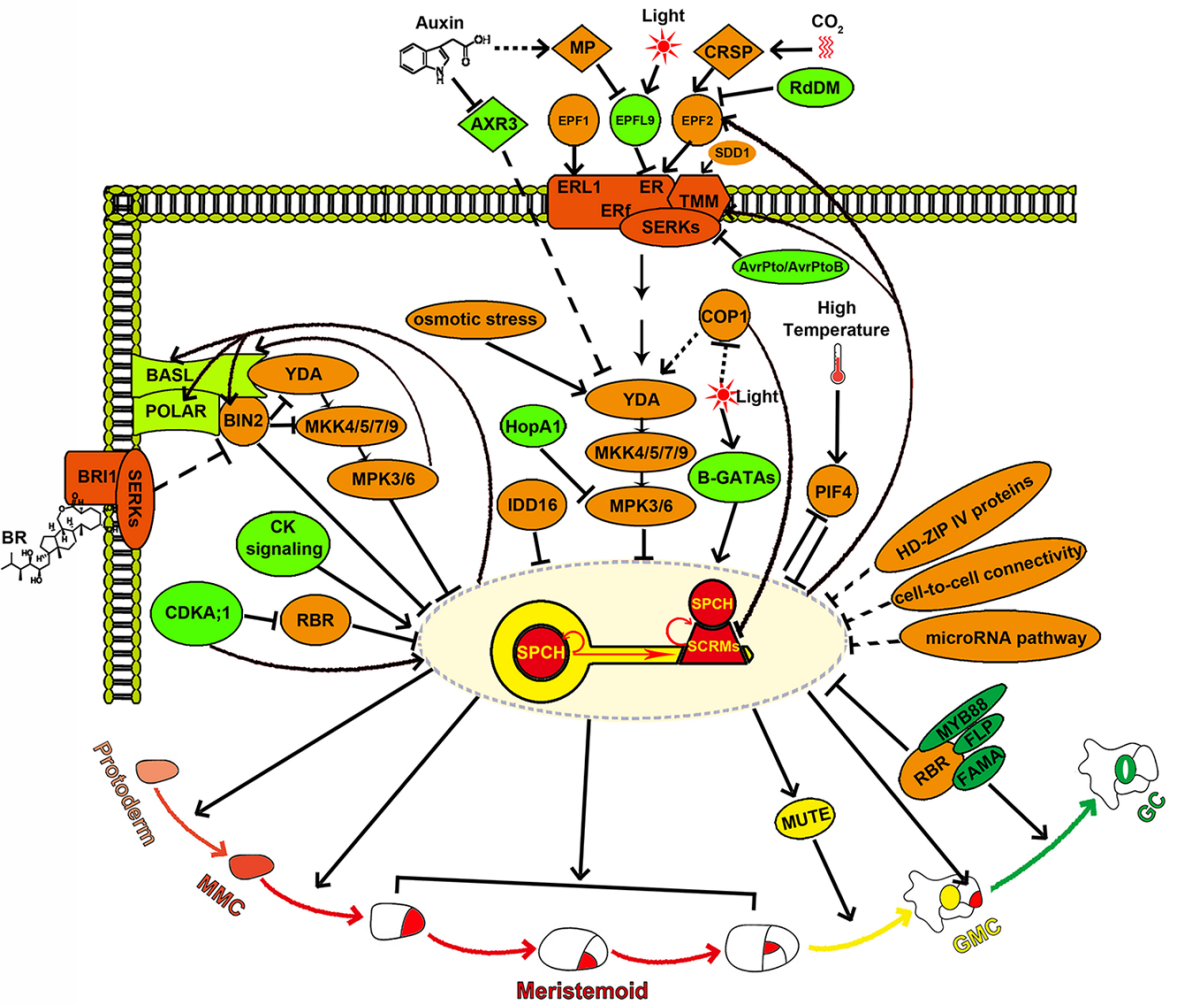
SPCH is the molecular key that opens stomatal development and acts as a central molecular hub while specifying stomatal cell fate. SPCH determines the entry into the stomatal lineage and integrates diverse developmental and environmental signals mediated by the YDA-MKK4/5/7/9-MPK3/6 cascade, BIN2, CDKA;1, B-GATAs, and PIF4. SPCH also directly regulates asymmetric cell division in the stomatal lineage through activating the transcription of the key polarity proteins BASL and POLAR. *SPCH* expression is tightly and spatiotemporally regulated by HD-ZIP IV proteins, cell-to-cell connectivity, microRNA pathway, IDD16, and RBR. SPCH enhances its own activity by activating itself and SCRMs, thereby maintaining the MMC and meristemoid fate, and suppresses itself by activating the EPF2-TMM signaling to ensure proper stomatal density and patterning.

A null stoma mutant named *spch-1* was identified in a sensitized genetic screen (MacAlister et al., 2007). *SPCH* encodes a bHLH transcription factor and has two closely related paralogues, *MUTE* and *FAMA*. *SPCH* is broadly transcribed in epidermal cells, but the SPCH protein is restricted to MMCs and meristemoids, suggesting that SPCH is strictly regulated at the posttranslational level (MacAlister et al., 2007). Closer observation showed that epidermal cells in *spch-1* did not undergo asymmetric entry division. In contrast, overexpression of *SPCH* induced ectopic entry division in the epidermis. These results suggest that *SPCH* is crucial for stomatal lineage initiation (**Figure 2**) (MacAlister et al., 2007; Pillitteri et al., 2007). The stomatal formation is also completely eliminated when both the two homologous bHLH-leucine zipper (bHLH-LZ) transcription factors, INDUCER OF CBF EXPRESSION1 (ICE1) and SCREAM2 (SCRM2), are knocked out (Kanaoka et al., 2008). Further research revealed that SPCH, MUTE, and FAMA heterodimerize with SCRMs (ICE1 and SCRM2) to trigger the successive MMC-meristemoid-GMC-GC fate transition (**Figure 2**) (Kanaoka et al., 2008). The direct targets of *SPCH* include *SPCH* itself and *ICE1*/*SCRM2*. SPCH and ICE1/SCRM2 can bind to their own promoters and enhance self-expression, thereby constituting a positive feedback loop that maintains the MMC and meristemoid fate (Lau et al., 2014; Horst et al., 2015) (**Figure 2**). In the grass *Brachypodium distachyon* and *Oryza sativa*, disabling either *SPCH* or *ICE1* eliminated stomata, suggesting that the *SPCH*/*ICE1* heterodimer also functions as a switch for the stomatal initiation in monocots (Raissig et al., 2016; Wu et al., 2019).

## SPCH Integrates Intralineage Signals for Proper Stomatal Density and Patterning

SPCH activity is inhibited by its phosphorylation and consequent degradation (Lampard et al., 2008). Interestingly, although the phosphorylation of SPCH is known to be mediated by mitogen-activated protein kinase 3/6 (MPK3/6), a direct interaction between MPK3/6 and SPCH has not been detected to date. A recent study has found that ICE1/SCRM2 acts as a scaffolding partner for their interaction (Lampard et al., 2008; Putarjunan et al., 2019). The direct association of MPK3/6 and ICE1/SCRM2 is also required for the phosphorylation and consequent degradation of ICE1/SCRM2, and this process is crucial for the proper specification of the stomatal cell fate (Putarjunan et al., 2019). Accordingly, a direct link between the SPCH•SCRM module and a MAPK cascade consisting of YODA (YDA), four MAPKKs (MKK4/5/7/9), and two MAPKs (MPK3/6) is established during the stomatal development (Bergmann et al., 2004; Wang et al., 2007; Lampard et al., 2009; Putarjunan et al., 2019). Upstream of the YDA-MKK4/5/7/9-MPK3/6 cascade lies a multiprotein receptor complex composed of the leucine-rich repeat receptor-like protein TOO MANY MOUTHS (TMM), the ERECTA family (ERf) leucine-rich repeat receptor-like kinases [ERECTA (ER), ERECTA-LIKE1 (ERL1), and ERECTA-LIKE2 (ERL2)], and SOMATIC EMBRYOGENESIS RECEPTOR KINASEs (SERKs) (Yang and Sack, 1995; Nadeau and Sack, 2002a; Shpak et al., 2005; Lee et al., 2012; Lee et al., 2015; Meng et al., 2015). These receptors can recognize several specifically expressed ligands that belong to the EPIDERMAL PATTERNING FACTOR-LIKE (EPFL) family of secreted cysteine-rich peptides to either repress or promote stomatal development in specific regions (**Figure 2**) (Hara et al., 2007; Hara et al., 2009; Hunt and Gray, 2009; Abrash and Bergmann, 2010; Hunt et al., 2010; Kondo et al., 2010; Sugano et al., 2010; Abrash et al., 2011; Lee et al., 2012; Niwa et al., 2013; Lee et al., 2015; Meng et al., 2015). EPF1, the first such peptide to be identified, is mainly dependent on ERL1 to ensure the correct spacing and meristemoid differentiation (**Figure 2**) (Hara et al., 2007; Lee et al., 2012). EPF2 is detected primarily by ER, which subsequently represses stomatal lineage initiation through the activation of the downstream MAPK cascade (Hara et al., 2009; Hunt and Gray, 2009; Lee et al., 2012; Lee et al., 2015). In contrast to EPF1 and EPF2, STOMAGEN/EPFL9 is a positive peptide that competes with EPF2 for ER association without triggering the downstream MAPK response (Hunt et al., 2010; Kondo et al., 2010; Sugano et al., 2010; Lee et al., 2015; Lin et al., 2017). In this way, STOMAGEN prevents the inhibitory activity of EPF2 (Lee et al., 2015) (**Figure 2**). In the stems, CHALLAH family peptides activate ERf receptors and inhibit stomatal development (Abrash and Bergmann, 2010; Abrash et al., 2011; Niwa et al., 2013). This ligand/receptor-mediated stomatal signaling pathway has also been reconstructed in mature *Nicotiana benthamiana* leaf cells (Jewaria et al., 2013). Epigenetic modifications on *EPF2* and *ERf* genes have been found to regulate stomatal development. The expression of *EPF2* is regulated by RNA-directed DNA methylation (RdDM), and the expression of *ERf* genes is regulated by histone modification and DNA methylation (Yamamuro et al., 2014; Wang et al., 2016). In addition, the subtilisin STOMATAL DENSITY AND DISTRIBUTION (SDD1), which is predicted to process peptide precursors that remain elusive, also acts upstream of TMM and YODA to repress stomatal formation (Berger and Altmann, 2000; von Groll, 2002; Lampard et al., 2008). The above intralineage signals are integrated by SPCH to regulate stomatal initiation and patterning. Moreover, *EPF2*, *TMM*, and *ERf* receptors are the direct targets of *SPCH* (Lau et al., 2014). SPCH and SCRMs directly activate the EPF2-TMM signaling, which in turn suppresses the SPCH•SCRM module, thus constituting a negative feedback loop that inhibits stomatal initiation and ensures the one-cell-spacing patterning (Lau et al., 2014; Horst et al., 2015) (**Figure 2**).

## SPCH Serves as an Acceptor of Hormonal and Environmental Signals to Regulate Stomatal Density and Patterning

SPCH directly integrates hormonal and environmental signals for stomatal formation. SPCH can be directly phosphorylated by the brassinosteroid (BR) signaling intermediate the glycogen synthase kinase 3 (GSK3)-like kinase BRINSENSITIVE 2 (BIN2), which is itself a direct target of SPCH, and this phosphorylation promotes the degradation of SPCH. Thus, BR promotes stomatal formation in hypocotyls though suppression of BIN2 mediated SPCH phosphorylation and degradation (Gudesblat et al., 2012; Yang et al., 2015). SPCH can also be directly phosphorylated by Cyclin-Dependent Kinases A;1 (CDKA;1). Unlike the negative regulation of SPCH by MAPK- and BIN2-mediated phosphorylation, CDKA;1 mediated phosphorylation of SPCH at Serine 186 promotes stomatal initiation, revealing that SPCH activity and stability are fine-tuned *via* phosphorylation by multiple kinases in response to various signals (Yang et al., 2015) (**Figure 2**). Increased cytokinin (CK) levels or signaling promotes SPCH expression, and SPCH directly induces the expression of the type-A ARABIDOPSIS RESPONSE REGULATOR16 (ARR16) and CLAVATA3/EMBRYO SURROUNDING REGION RELATED 9/10 (CLE9/10) (Lau et al., 2014; Vaten et al., 2018). ARR16 negatively regulates CK response and CLE9/10 represses type-A ARRs. The SPCH-dependent activities of the repressive type-A ARR16/17 and the secreted peptides CLE9/10 are essential for establishing local domains of low CK signaling, which inhibits both SLGC division and stomatal formation (Vaten et al., 2018). ARR16/17 and CLE9/10 counteract the proliferative effect of SPCH to customize the epidermal cell-type composition (Vaten et al., 2018). CLE9/10 peptides are also recognized by the receptor kinase HAESA-LIKE 1 (HSL1) to regulate the stomatal lineage cell division; however, the underlying mechanism is unknown (Qian et al., 2018). The heat-stress signaling induces the accumulation of PHYTOCHROME-INTERACTING FACTOR 4 (PIF4) in stomatal precursors. PIF4 can directly bind to *SPCH* and repress its expression, while the SPCH protein, in turn, inhibits the expression of *PIF4*, thus producing a negative feedback loop to control stomatal development in ﬂuctuating temperatures (Lau et al., 2018). Red light can induce the expression of both *SPCH* and GATA factors of the B-subfamily (B-GATA) transcription factors. B-GATAs directly bind to the *SPCH* promoter and are required for the red-light-dependent induction of *SPCH* expression (Klermund et al., 2016).

SPCH also serves as a final acceptor of hormonal and environmental signals accepted by its upstream signaling factors. BR has also been shown to inhibit stomatal formation in the leaf epidermis through the inactivation of BIN2. In this scenario, BIN2 has been found to repress YDA and MKK4/5 activation, promoting SPCH stabilization (Kim et al., 2012; Khan et al., 2013) (**Figure 2**). Another phytohormone, auxin, negatively regulates stomatal formation partially by activating auxin response factor 5 (ARF5) and inhibiting AUXIN RESISTANT3 (AXR3). ARF5 suppresses stomatal formation by directly repressing STOMAGEN expression in the mesophyll, while AXR3 promotes stomatal production by functioning upstream of the YDA MAPK cascade in dark-grown seedlings (Balcerowicz et al., 2014; Le et al., 2014; Zhang et al., 2014) (**Figure 2**). Light signals are perceived by multiple photoreceptors to promote stomatal formation by inhibiting the RING E3 ubiquitin ligase CONSTITUTIVE PHOTOMORPHOGENIC 1 (COP1) (Lau and Deng, 2012). COP1 acts genetically upstream of YDA to repress the stomatal development and can also stimulate the degradation of SCRM proteins through ubiquitin/proteasome pathways in the dark (Kang et al., 2009; Lee et al., 2017) (**Figure 2**). In addition, increased light irradiation increases stomatal density by inducing the expression of *STOMAGEN* (Hronkova et al., 2015) (**Figure 2**). Elevated atmospheric carbon dioxide (CO_2_) levels induce the expression of *CO2 RESPONSIVE SECRETED PROTEASE* (*CRSP*), and the encoded protein can cleave the pro-peptide EPF2 (**Figure 2**). Thus, high concentrations of CO_2_ may repress stomatal formation primarily by the EPF2-mediated negative regulation pathway (Engineer et al., 2014). Osmotic stress decreases stomatal number by downregulating SPCH protein level. This process is mediated by the MAPK-SPCH core developmental pathway (Kumari et al., 2014) (**Figure 2**). Stomata also serve as bacterial entry gates (Melotto et al., 2006; Melotto et al., 2017). The pathogen *Pseudomonas syringae* invades hosts through stomatal pores and releases the effector HopA1 (Melotto et al., 2006; Zhang et al., 2007). Overexpression of HopA1 in plant specifically inactivates MPK3/6, leading to stomatal clustering (Kim et al., 2012) (**Figure 2**). In addition, the inducible overexpression of AvrPto and AvrPtoB, two effector proteins of *P. syringae pv. tomato* (*Pst*), also generates clustered stomata in *Arabidopsis* (Meng et al., 2015). AvrPto and AvrPtoB may promote stomatal formation through impairing the function of their target SERKs, which act as coreceptors along with the ER-TMM complex (**Figure 2**).

## SPCH Regulates Asymmetric Cell Division in the Stomatal Lineage

SPCH induces the expression of *BREAKING OF ASYMMETRY IN THE STOMATAL LINEAGE* (*BASL*) and *POLAR* in the stomatal lineage. Both BASL and POLAR proteins exhibit a polarized peripheral localization during the stomatal lineage asymmetric cell division (ACD). Phosphorylation of BASL by MPK3/6 enhances its interaction with YDA, leading to the recruitment of YDA to the cell cortex (Dong et al., 2009; Zhang et al., 2015; Zhang et al., 2016). Thus, BASL serves as a scaffold protein that spatially concentrates MAPK signaling in the cortex and segregates MAPK signaling into SLGCs after ACD (Zhang et al., 2015). The enhanced YDA-MPK3/6 signaling in SLGCs promotes the phosphorylation and degradation of SPCH, leading to the differentiation of SLGCs into pavement cells. However, the low level of YDA-MPK3/6 signaling in meristemoids results in stable SPCH expression, triggering the subsequent developmental processes (Zhang et al., 2015). POLAR polarization requires BASL activity (Pillitteri et al., 2011), and POLAR appears to function together with BASL to regulate the stomatal lineage ACD by confining BIN2 to the cell cortex (Houbaert et al., 2018). This regulation can relieve the inhibition of SPCH by BIN2, thus freeing SPCH to drive ACD (Houbaert et al., 2018).

## *SPCH* Expression Is Tightly and Spatiotemporally Regulated

The *HOMEODOMAIN LEUCINE ZIPPER CLASS IV* (*HD-ZIP IV*) family genes *MERISTEM LAYER 1* (*ML1*) and *HOMEODOMAIN GLABROUS2* (*HDG2*) function in establishing and maintaining epidermal identity. Their ectopic expression induces the formation of ectopic epidermal layers with *SPCH* expression and stomatal formation in internal leaf tissues, suggesting that the acquisition of epidermal layer identity is required for *SPCH* expression and stomatal lineage fate (Peterson et al., 2013; Takada et al., 2013).

Plasmodesmatal permeability and cellular integrity in the epidermis confine SPCH to stomatal lineage cells during stomatal development (**Figure 2**). Mutating the callose synthase *GLUCAN SYNTHASE-LIKE 8* (*GSL8*/*CHORUS*) or the glycosyltransferase-like protein KOBITO1 disrupts cellular integrity or increases plasmodesmata permeability. These defects allow intercellular movement of SPCH protein in the leaf epidermis, resulting in clustered stomata formation and disorganized cell divisions in the stomatal lineage (Guseman et al., 2010; Kong et al., 2012).

A microRNA pathway is presumed to repress stomatal lineage initiation through regulating *SPCH* transcripts (**Figure 2**) (Kutter et al., 2007; Yang et al., 2014). In addition, IDD16, a C2H2 zinc finger transcription factor from the INDETERMINATE DOMAIN (IDD) family, and RETINOBLASTOMA RELATED (RBR), which is targeted by CDKA;1, have been shown to inhibit stomatal initiation by directly binding to *SPCH* and repressing *SPCH* transcription (**Figure 2**) (Weimer et al., 2012; Qi et al., 2019). The specific downregulation of *RBR* in GMCs and GCs leads to excess divisions in differentiated GCs and formation of the “Stoma-in-Stoma” (SIS) phenotype (Lee et al., 2014b; Matos et al., 2014). Histone3 K27 trimethylation (H3K27me3) is involved in maintaining the GC identity (Lee et al., 2019), and its reduced deposition on the *SPCH* and *MUTE* loci is responsible for the SIS phenotype (Lee et al., 2014b; Matos et al., 2014). Consistent with this, constitutive expression of CURLY LEAF (CLF), a member of Polycomb Repressive Complex 2 (PRC2) that functions in H2K27me3 and other chromatin modiﬁcations, suppresses the SIS phenotype (Lee et al., 2014b). RBR has been shown to interact with PRC2, FAMA, and FLP/MYB88, which redundantly functions with FAMA to inhibit GMC division (Desvoyes et al., 2010; Magyar et al., 2012; Lee et al., 2014a). Both RBR and FAMA target the promoters of *SPCH*, *EPF1*, and *FAMA* (Matos et al., 2014). Thus, a model in which RBR and the PRC2 components are recruited by FAMA to the promoters of *SPCH* and other stomatal lineage genes has been presented. This complex represses the re-expression of those genes and the reinitiation of stomatal lineage through chromatin modification (Matos et al., 2014).

## Conclusion and Perspective

In summary, SPCH acts as a central molecular hub that integrates both developmental and environmental signals while specifying stomatal cell fate. However, many questions remain to be addressed. Firstly, more external and internal cues that are integrated into the SPCH node need to be identified to further understand how stomatal development adjusts to a fluctuating environment. Secondly, although SPCH mostly functions upstream of the stomatal lineage, little is known about how SPCH transcription is initiated and regulated. In addition, although the direct target genes of SPCH have been known for years, most of their functions remain elusive. Lastly, RNA polymerase II (Pol II) is essential for stomatal patterning and differentiation (Chen et al., 2016), and it is unknown how SPCH recruits Pol II for specific gene expression. SPCH is the core regulator of stomatal density. Genetic manipulation of stomatal density to improve plant productivity and water consumption efficiency has been proven to be feasible in barley and rice (Hughes et al., 2017; Caine et al., 2019). Future studies focusing on the above questions will provide invaluable potential targets for genetic improvement of agriculturally relevant species to promote sustainable agricultural development.

## Author Contributions

LC wrote the manuscript, drew figures, and edited its final form. ZW contributed critical evaluation of the text. LC and SH conceived the topic.

## Funding

This work was supported by National Natural Science Foundation of China (NSFC) (grant numbers 31670185, 31870251, 31800237); the Ministry of Agriculture of the People's Republic of China (grant number 2016ZX08009003-002); the major project of Science and Technology of Gansu province (grant number 17ZD2NA016-5, 17ZD2NA015-06); China Postdoctoral Science Foundation Grant (grant number 2017M620478); and the Chang Jiang Scholars Program of China (2017).

## Conflict of Interest

The authors declare that the research was conducted in the absence of any commercial or financial relationships that could be construed as a potential conflict of interest.
